# A case report of recalcitrant aphthous ulcers in two patients treated with interleukin-17 inhibitors

**DOI:** 10.1177/2050313X211034925

**Published:** 2021-09-03

**Authors:** Madison Ward, Khalad Maliyar, Melinda Gooderham

**Affiliations:** 1 SKiN Centre for Dermatology, Peterborough, ON, Canada; 2 University of Toronto, Toronto, ON, Canada; 3 Probity Medical Research, Waterloo, ON, Canada; 4 Queen’s University, Kingston, ON, Canada

**Keywords:** Interleukin-17, psoriasis, Behçet’s disease, aphthous ulcers, ulcers

## Abstract

The interleukin-17 signaling pathway plays a critical role in the pathogenesis of psoriasis. By inhibiting this pathway, there is a reduction in the severity of psoriasis and many patients achieve clear skin. We present two individuals, a 34-year-old male and a 40-year-old female, who developed aphthous ulcers on the oral mucosa and oral and vulvar mucosa, respectively, while undergoing treatment with interleukin-17 inhibitors. The ulcers did not respond to conventional therapy, including topical corticosteroids and nystatin 100,000 unit/mL oral suspension. Both patients underwent biopsies which confirmed the diagnosis of ulcer. Once confirmed, the interleukin-17 inhibitor was discontinued and the ulcers resolved in both cases. As we see biologic treatment, specifically interleukin-17 inhibitor, becoming more popular for the treatment of psoriasis, it is important for physicians to be aware of this potential adverse event. Early detection and intervention are important to avoid complications that can develop from rare but often painful ulcers.

## Introduction

In psoriasis, there is a dysregulated innate and adaptive immune system, whereby interleukin (IL)-17, a proinflammatory cytokine produced by T helper 17 (Th 17) cells, causes keratinocyte hyperproliferation and vascular response changes.^1^ Several monoclonal antibodies targeting the IL-17 pathway have been approved by both the Health Canada and the US Food and Drug Administration (FDA), including ixekizumab and secukinumab targeting the IL-17A cytokine, and brodalumab targeting the IL-17RA receptor subunit. These antibodies have been shown through Phase 3 clinical trials to have significant clinical efficacy in reducing the severity of psoriasis, as well as providing an overall favorable safety profile.^2–4^ The most common adverse events associated with these biologic agents include infections, nasopharyngitis, and headache.^2–4^ There have been post-marketing reports of adverse events including inflammatory bowel disease,^5^ mucosal lichenoid reactions,^6–8^ and Behçet’s disease.^9,10^

## Case report

We report two cases of individuals who developed recalcitrant aphthous ulcers while undergoing IL-17 inhibitor therapy for psoriasis.

**Figure 1.**
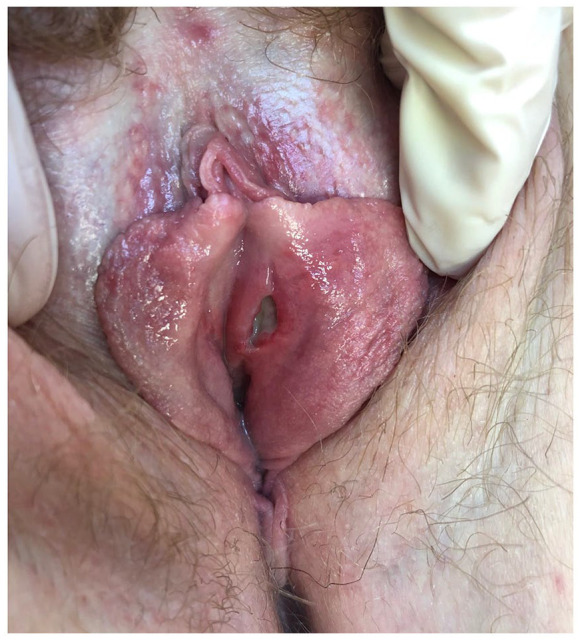
Case #1 with deep vulvar ulcer while receiving secukinumab.

First, we report a 40-year-old woman who has had psoriasis since 16 years of age. She was otherwise healthy and not on any routine medications other than topical steroids for her psoriasis. After failure of both topical steroids and phototherapy, she began subcutaneous secukinumab 300 mg label dose in May 2017 to treat her psoriasis with an initial good response. Due to losing response, her dose was increased to 300 mg Q3 weeks in early 2019 and then again to 300 mg Q2 weeks in May 2019. She presented to the clinic in August 2019 with an 8-month history of recurrent oral ulcers and a 1-month history of a deep painful vulvar ulcer (see Figure 1). After she failed a course of anti-viral therapy prescribed by her family doctor and had two negative vulvar viral swabs for herpes simplex virus as well as a negative polymerase chain reaction (PCR) test for *Haemophilus ducreyi*, a biopsy was performed, and her family doctor asked her to hold secukinumab. The histopathology showed an ulcerated lesion with a differential diagnosis of either viral infection, aphthous ulcer or Behçet’s disease. A decision was made to discontinue the secukinumab and treat the ulcers symptomatically with a topical steroid and a topical anesthetic, at which point she experienced a flare up of her psoriasis. The patient was started on risankizumab, an IL-23 inhibitor, for her psoriasis. When presenting for her first risankizumab injection, she was noted to have dactylitis of her second digit and a referral to Rheumatology was made. She was given a new diagnosis of psoriatic arthritis which was also managed by risankizumab. Her vulvar ulcer completely healed 4 months after the discontinuation of the secukinumab. Since starting risankizumab, she has not had recurrence of any oral or vulvar aphthous ulcers, her psoriasis remains clear, and her joint disease was controlled after 16 months of follow-up.

**Figure 2.**
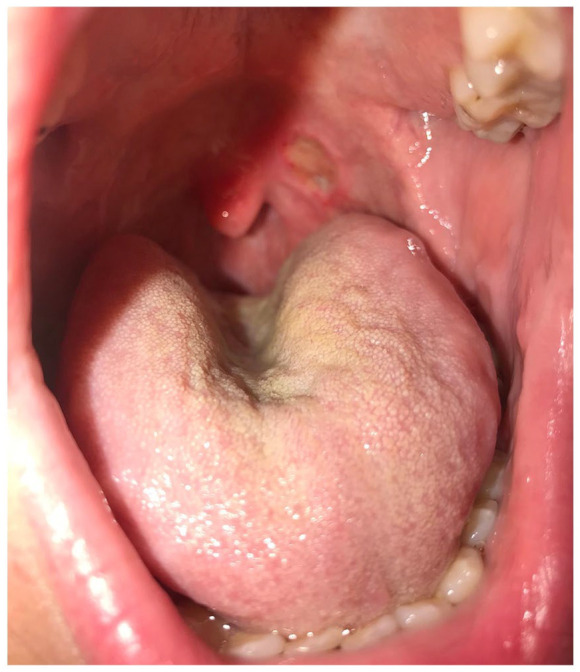
Case #2 persistent oral ulcer while receiving ixekizumab.

Second, we report the case of a 34-year-old man who has a long history of severe plaque psoriasis. He also has a medical history of psoriatic arthritis, obesity, cholelithiasis, and multi-level disk herniation. He had been on multiple treatments over the years for his psoriasis including topical steroids, phototherapy, methotrexate (2005), brodalumab (2012–2015), cyclosporine (2015), and ixekizumab (2016–2019). In May 2019, the patient presented with a 3-week history of a painful oral ulcer that was not responding to topical anti-fungal therapy (see Figure 2). His ixekizumab was held while he was referred to Otolaryngology for work up. He had multiple biopsies which suggested an aphthous ulcer. The first biopsy showed a mucosal ulcer with adjacent reactive changes and had no evidence of fungal organisms on periodic acid–Schiff (PAS) stain, but the second had features suggestive of superficial fungal infection. A third biopsy for direct immunofluorescence was negative. He did not respond to repeated courses of nystatin 100,000 unit/mL swish and swallow oral suspension and topical corticosteroid. After discontinuing ixekizumab therapy and switching to risankizumab in September 2019, his ulcer healed, and he has not had a recurrence after 17 months of follow-up. His skin has remained controlled and joint disease activity has been stable since the switch to risankizumab therapy.

## Discussion

Aphthous ulcers are the most prevalent cause of recurrent ulcers of the mucous membranes. They arise as a result of dysfunction in the regulation of cellular immunity that leads to increased T-cell mediated damage to mucous membrane keratinocytes. This abnormal response by the immune system is believed to be triggered by many factors including hormonal fluctuations, psychological stress, trauma, and iron and vitamin B_12_ deficiencies. Differential diagnosis includes erosive lichen planus, herpangina, hand-foot-and-mouth disease, Behçet’s disease, and mucous membrane pemphigoid.^11^

There are three morphologic types of aphthous ulcers of which to be aware. Minor aphthae are single to multiple, shallow, mildly painful ulcers that are less than 1.0 cm in diameter. They heal within 1–2 weeks without any scarring. Major aphthae are larger ulcers that are 1–3 cm in diameter, deeper, and can persist from 2 to 6 weeks. They heal with scarring and can be associated with systemic symptoms, including fever, malaise, and odynophagia. Finally, herpetiform aphthae are uncommon and consist of multiple painful oral ulcerations that are 1–3 cm in diameter, have a similar clinical course to minor aphthous ulcers, and superficially resemble primary herpes simplex virus infection.^12^

These ulcers are diagnosed clinically, but a biopsy can be performed to rule out other causes. In this case, we wanted to investigate for other causes related to IL-17 inhibition, such as candidiasis or cutaneous Crohn’s disease. Histopathology results of aphthous ulcers are typically non-specific, but early lesions can demonstrate a neutrophilic vessel–based submucosal infiltrate. Treatment options include topical agents for symptomatic relief such as superpotent topical corticosteroid gels, topical analgesics, or intralesional corticosteroids. Systemic therapies such as colchicine, dapsone, or thalidomide can be used for more severe cases.^13^

Although IL-17 inhibitors have an overall acceptable safety profile, there have been several recent reports of paradoxical adverse reactions of the oral and genital mucosa, not expected according to their mechanism action. A number of oral lichenoid reactions have been reported in the literature to date. Capusan et al.^6^ described a psoriatic patient with an oral lichenoid reaction of the tongue that developed 8 months after starting secukinumab therapy. Secukinumab has also been reported to induce oral lichen planus associated with candidiasis, 5 months after beginning secukinumab therapy for psoriasis.^7^ Another male with erythrodermic psoriasis developed a painful ulcerative lichenoid mucositis of the lower lip several days after initiating secukinumab therapy.^8^ Secukinumab has also been implicated in both the exacerbation and de novo emergence of Behçet’s syndrome for two individuals with ankylosing spondylitis treated with secukinumab.^10^ Shiga et al.^9^ reported a case of inflammatory bowel disease and Behçet’s after the use of secukinumab to treat psoriasis.

We describe two patients who presented with persistent ulcerations of the oral mucosa and oral and vulvar mucosa, which did not respond to conventional treatment with topical steroids, topical anti-fungals, and systemic anti-viral agents. Biopsies confirmed aphthous ulcers and ruled out other diagnoses. Discontinuation of the IL-17 inhibitors and switching to the IL-23 inhibitor, risankizumab, allowed the ulcers to heal with no recurrence after more than 1 year of follow-up. As IL-17 inhibitors are increasingly used for the treatment of psoriatic disease, it is important that these events are reported. Physicians prescribing drugs from this class should be aware of these rare, but possible adverse events upon administration as early detection and treatment can help decrease the morbidity of these often painful lesions.
